# Supplementary material for: A high-resolution mRNA expression time course of embryonic development in zebrafish
Source: eLife. 2017 Nov 16;6:e30860. doi: 10.7554/eLife.30860 (PMC5690287; doi:10.7554/eLife.30860)
Supplement: Supplementary file 6. [file elife-30860-supp6.zip › biolayout-clusters-files/Cluster121-genes.html]

Cluster121


# Cluster121: Genes

| Ensembl ID | Gene Name | Chr | Start | End | Biotype |
| --- | --- | --- | --- | --- | --- |
| ENSDARG00000100022 | FO704797.2 | 17 | 12980980 | 13001704 | protein\_coding |
| ENSDARG00000028517 | hbp1 | 25 | 3325980 | 3345373 | protein\_coding |
| ENSDARG00000079840 | kcnma1a | 13 | 16390976 | 16758119 | protein\_coding |
| ENSDARG00000017612 | pnpo | 12 | 28685086 | 28692282 | protein\_coding |
| ENSDARG00000101215 | sgip1b | 2 | 14608049 | 14722037 | protein\_coding |
| ENSDARG00000103802 | si:ch73-389b16.2 | 6 | 24053007 | 24104412 | protein\_coding |
| ENSDARG00000075382 | slc9a6b | 10 | 26609461 | 26635278 | protein\_coding |
| ENSDARG00000025011 | synj1 | 10 | 26554233 | 26607637 | protein\_coding |
| ENSDARG00000005670 | ttbk2 | 17 | 45102319 | 45133567 | protein\_coding |
| ENSDARG00000067832 | zgc:136336 | 2 | 52732447 | 52792114 | protein\_coding |
| ENSDARG00000079847 | zgc:194578 | 19 | 10460323 | 10476758 | protein\_coding |
